# Skeletal muscle as an experimental model of choice to study tissue aging and rejuvenation

**DOI:** 10.1186/s13395-020-0222-1

**Published:** 2020-02-07

**Authors:** Jessy Etienne, Chao Liu, Colin M. Skinner, Michael J. Conboy, Irina M. Conboy

**Affiliations:** Department of Bioengineering and QB3 Institute, University of California, Berkeley, Berkeley, CA 94720-3220 USA

**Keywords:** Aging, Myogenesis, Stem cells, Niche, Tissue repair, Inflammation, Signaling pathways, Epigenome, Satellite cells, Rejuvenation

## Abstract

Skeletal muscle is among the most age-sensitive tissues in mammal organisms. Significant changes in its resident stem cells (i.e., satellite cells, SCs), differentiated cells (i.e., myofibers), and extracellular matrix cause a decline in tissue homeostasis, function, and regenerative capacity. Based on the conservation of aging across tissues and taking advantage of the relatively well-characterization of the myofibers and associated SCs, skeletal muscle emerged as an experimental system to study the decline in function and maintenance of old tissues and to explore rejuvenation strategies. In this review, we summarize the approaches for understanding the aging process and for assaying the success of rejuvenation that use skeletal muscle as the experimental system of choice. We further discuss (and exemplify with studies of skeletal muscle) how conflicting results might be due to variations in the techniques of stem cell isolation, differences in the assays of functional rejuvenation, or deciding on the numbers of replicates and experimental cohorts.

## Background

**Fig. 1:**
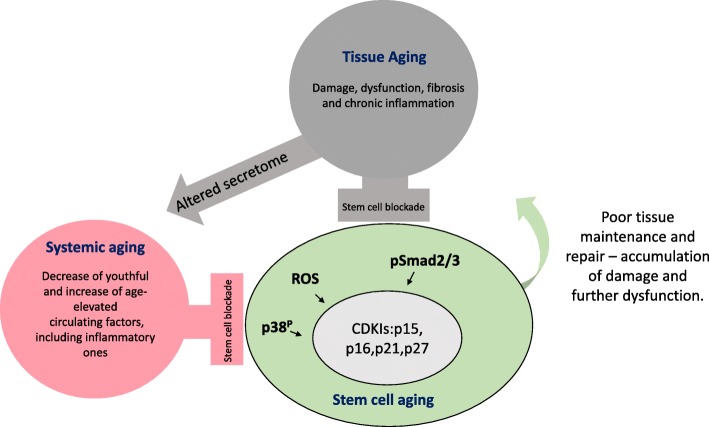
Fundamental theory of progressive tissue aging that fits with the phenomena of rapid experimental rejuvenation. Increasing with chronological age, damage to differentiated soma – tissue niches of stem cells blocks regenerative responses through deregulation of cell-niche crosstalks. With worsened regenerations, tissues become more damaged (increase in inflammation and fibrosis) and their secretome changes thereby altering the composition of systemic milieu, affecting tissues at a distance, and further inhibiting the capacity of adult stem cells to maintain and repair the tissues

Several theories of aging have been proposed: cellular senescence [1], accumulation of mutations [2], antagonistic pleiotropy [3], disposable soma [4], deteriorated proteostasis [5], or telomere attrition [6]. While relevant and valid in many instances, each of these theories alone does not explain the rapid and robust rejuvenation of old tissues observed in heterochronic parabioses and blood exchange studies [7–11]. An alternative theory that fits both the aging and the rejuvenation data [12] suggests that aging is caused primarily by the functional (and notably, experimentally reversible) inactivation of resident stem cells, which precipitates deteriorated tissue maintenance and repair and leads to the loss of organ homeostasis [13]. The damaged and unrepaired tissues suffer changes in their biochemistry, including the molecular crosstalk with resident stem cells, which further inhibits productive, regenerative responses. The inflammatory and fibrotic secretome can then propagate systemically, affecting the entire organism [10, 14–23]. This decline in homeostatic functional integrity causes age-associated diseases, the degenerative and inflammatory disorders of the muscle, brain, liver, and bone, diminished immune responses, and increased susceptibility to infections, cancers, cardiovascular diseases, and metabolic diseases (e.g., type II diabetes) [24]. Figure 1 illustrates the above-introduced theory of aging.

Skeletal muscle (note that “muscle” does not include smooth and cardiac muscle in this review) accounts for almost 40% of the total adult human body mass. This tissue is indispensable for vital functions such as respiration, locomotion, and voluntary movements and is among the most age-sensitive in mammals. Aging muscle loses its ability to adapt its morphological, biochemical, biophysical, and molecular properties to loads and use. With advanced age, interventions such as exercise do not efficiently reverse the rapid loss of muscle mass resulting from disuse atrophy and systemic diseases. Numerous age-associated changes have been investigated: fiber atrophy [25–27], increase in apoptosis [28], DNA damage [29, 30], heterochromatin marks [31], reduced protein synthesis [32, 33], autophagic degradation [34], lysosomal dysfunction characterized by lipofuscin accumulation [35, 36], accumulation of advanced glycation end-products [37], insoluble polyubiquitylated proteins [38], changes in microRNA expression [39], and altered nuclear shape and spatial disorganization of nuclei [40]. These age-specific parameters are not unique to muscle and manifest more generally, in other organs and tissues, such as the immune system, CNS, bone, skin, and liver [41, 42]. Similarly, the decline in numbers and functional activation seen with muscle satellite cells (SCs) are also seen in other tissues such as blood, brain, bone, and liver [41, 42]. The age-specific changes in the resident stem cell pools diminish the regenerative potential that is needed to compensate for tissue loss due to attrition or injury. As typical of tissue aging, the aged muscle becomes infiltrated by adipose tissue and fibrosis, shows decreased capillarization, and is characterized by chronic inflammation. Altogether, these changes result in a progressive reduction in myofiber size and number that collectively are seen as a progressive decline in muscle mass, cross-sectional area, and strength, a phenomenon known as sarcopenia.

Muscle is relatively accessible for ectopic gene expression, given that it is a non-vital tissue with a good ability to uptake gene constructs after single or repeated injections into the tissue or through systemic delivery. Using screens for native gene expression and gene reporters, the markers and biochemical regulators of SCs have been identified and characterized [43]. Additional methods, including tissue histology, biochemistry, cell isolation and characterization by function, and gene expression-omics studies, have allowed decrypting age-specific SCs properties, changes in differentiated myofibers, and the dynamics between SCs and their muscle niches. The SCs niche controls the maintenance and breakage of quiescence, decisions to self-renew or differentiate, and asymmetric versus symmetric divisions. In SCs, chromatin adopts bivalent states to facilitate rapid differentiation in response to external factors, and metabolism adapts to support particular needs. Stem cell niche control of SCs is age-specific and is generally conserved between adult tissue stem cells [41, 42].

This review summarizes current approaches that used skeletal muscle for improving our understanding of the crosstalk between adult stem cells and their niches, which, when altered by aging, leads to reduced tissue maintenance and repair. We also discuss how tissue rejuvenation might be pursued. We further elaborate on differences in the experimental design in the field of aging and rejuvenation that might have led to conflicting results, and we point out critical steps for ensuring robust experimental outcomes.

### Life-long stem cell persistence, age-specific dysfunction, and loss of heterogeneity

Muscle is capable of active repair in response to daily wear and tear, intense exercises, or injuries. Unfortunately, there is a noticeable decline in muscle regeneration and performance after 40 years, and this tissue becomes typically dysfunctional after the seventh decade, characterized by severe loss of muscle mass or sarcopenia [44–48]. Muscle regeneration relies on the adult muscle stem cells, also called satellite cells (SCs) due to their location around the periphery of the sarcolemma, under the basal lamina of each mature myofiber. Decades of studies have provided abundant information on the SC markers, tissue location, signaling pathways that control their function, and the age-imposed changes in any of the above [7, 8, 49–53].

**Fig. 2:**
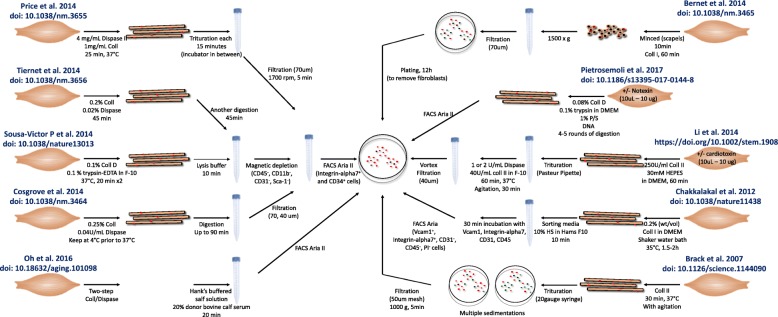
Variation in isolation of heterogeneous tissue stem cells. Illustrated are the different methods of satellite cell isolation, which all have been used in studies of muscle aging and rejuvenation. Considering that satellite cells (and tissue stem cells in general) are a heterogeneous population, enrichments for different sub-populations produce results and conclusions that might fail to apply broadly to the entire stem cell pool and might differ from lab to lab

The inherent heterogeneity of the SC pool might have led to conflicting results in the aging field because different groups employ different approaches for SCs identification and isolation (summarized in Fig. 2), thus analyzing different subsets of the heterogeneous population which have different properties. Historically, SCs were first identified and studied in muscle cryosection by electron microscopy [54] and are currently studied through immuno-fluorescence imaging. Since their first observation in the tibialis anticus (anterior) muscle of the frog [54], several markers have allowed SCs identification in many animals: human, mouse, monkey, pig, chick, salamander, frog, and zebrafish [55–57]. These adult stem cell markers include Barx2 [58], c-Met [59], calcitonin receptor [60], caveolae-forming protein caveolin 1 [61], CD34 [51, 62], CD56 [63, 64], CXCR4 [65, 66], Emerin [61], Lamin A/C [40], M-Cadherin [51], NCAM [67], Notch1 [67], VCAM1 [68], Pax3 [69], syndecan3 [70], syndecan4 [67, 70], and Sca1 [66], but by far, Pax7 [71] is the most widely used and evolutionarily conserved SC marker for fetal and adult SCs [72].

Most of the studies investigating aged SCs properties (e.g., proliferation and differentiation capacities) use fluorescence-activated cell sorting (FACS) on the broadly expressed CXCR4, CD34, or additional myogenic markers (e.g., M-Cadherin, alpha7-integrin, syndecan4, VCAM1 and ITGB1), while negatively selecting against CD45 leukocytes, CD31 endothelial cells, and Sca1-expressing cells. Cell sorting can be damaging for cell viability and function and, more importantly, enriches for a sub-population of SCs, both focusing on that population and yet limiting the study only to that subset [53, 66, 68, 73–75]. Alternative methods, such as density gradient purification, requires multiple centrifugations and also can compromise cell viability and function and require high starting cell numbers, thus calling for experimental injury by myotoxins or cardiotoxin, or expansion of the cells in culture, thus allowing further deviation from in vivo properties and gene expression [76].

Methods that do not limit the study to a subset consist of chopping the muscle into small pieces and, after mesh filtration and/or pre-plating on plastic culture dishes, expansion of the fewer adherent cells in Ham’s F-10 Nutrient Mixture (F-10), 20% FBS, 2.5–5 ng/ml bFGF [52, 77]. While in this bulk preparation, no sub-population is excluded, SCs are contaminated with other cells, including fibroblasts, endothelial cells, and macrophages. Such contamination with irrelevant cell types may be minimized by the culture of single myofiber explants or two-step enzymatic dissociation of myofibers with their associated SCs. The type of enzyme depends on the species and digestion methods [78–80], but after removal of the more adherent fibroblasts (for instance, by pre-plating on uncoated tissue culture dishes), the SC myogenic pool reaches 95–99% of purity and the stem cell properties, gene expression, and heterogeneity are preserved [78, 81–87].

Within the muscle, around 85% of SCs are located in proximity to blood vessels [88], and these cells display heterogeneities of metabolism, the ability for long-term renewal versus differentiation, and expression of Pax7 or Myf5. Quiescent SCs exist as a continuum from Pax7^low^ cells that are primed for cell-cycle entry to Pax7^high^ cells that are in deeper state of quiescence [89]. The number of SCs varies by muscle types, and overall declines with age [90–95], although whether this decline is slight or severe is a matter of some debate [10, 14, 51, 52, 96–98]. The hindlimb muscles of newborn and juvenile rodents contain a mix of SCs and their more differentiated progeny: proliferating myoblasts that are numerous, summing to around 30% of total sublaminar myonuclei, and supporting the rapid growth of juvenile muscle. When a more quiescent adult stem cell pool is established in 2-month-old mice [99–101], the SCs represent less than 5% of myofiber sublaminar nuclei and remain relatively constant in adulthood. Adult muscle is hence composed of postmitotic multinucleated myofibers and their associated non-dividing, quiescent SCs. By a geriatric 30 months of age, SCs represent 2.5% of the total muscle cells [71, 102, 103]. Yet this decline is not drastic compared to adult or old mice when normalized to muscle mass, which has also declined by such an advanced age [10, 14, 51]. Another important variable to account for when determining the number of SCs is the muscle type. Generally, adult slow-twitch myofibers (type I) such as that predominate in the soleus are generally associated with two- to fourfold higher SC numbers than fast-twitch, type IIa and IIb myofibers that predominate in the tibialis anterior or EDL [104].

SCs are critically needed for the regeneration of injured muscle fibers and, to a small extent, they participate in the process of overload hypertrophy, for example when muscle fibers grow through protein synthesis and become bigger there might be some SC proliferation to populate the enlarged fiber mass [105–107]. Conversely, muscle fibrosis and atrophy can be induced by SC depletion [108–111]. Cellular homeostasis is tightly regulated in muscle, as evidenced from the restoration of sufficient quiescent SCs after a local tissue injury, to support future needs of repair [112, 113]. Rather than a significant decline in the total number with age, most of the data support a dramatic lack of activation of muscle stem cells after injury and a concomitant lack in the formation of progenitors that are needed for repair [7, 8, 114, 115]. This lack of myogenic cells is in part due to reduced asymmetric divisions among myogenic stem and progenitor cells and is also linked to diminished SC self-renewal [53, 116–118].

### Age-specific changes in key signaling pathways

Signaling pathways play essential roles in SC maintenance and adult myogenesis, which largely recapitulates the cellular and molecular regulations that take place during embryonic myogenesis. Notch signaling plays a critical role by regulating the quiescence and proliferation decisions of SCs, in cooperation with syndecan3, and in influencing asymmetric cell division through antagonism with the Wnt/beta-catenin signaling. Notably, the age-specific role of Notch and Wnt interplay, as well as that of the TGF-beta, Jak/Stat, etc. pathways that was deciphered in muscle, is conserved in the brain, blood, bone, gut, and other tissues [119–122].

The Notch ligand Delta1 is upregulated by damaged myofibers and provides the temporal and positional cues for Notch activation in quiescent SCs [7, 49, 51]. Notch signaling promotes myoblast proliferation and inhibits their differentiation [49, 51, 123–126] in part through antagonism with Wnt signaling [50]. Notch also contributes to return of Pax7+MyoD- cells to quiescence [127]. Muscle regeneration relies on the tight balance between self-renewal and myogenic commitment. With age, SCs undergo excessive commitment and precocious differentiation [52], revealing a dysfunction in the ability to undergo proper asymmetric division. Delta expression and hence Notch activation is lacking in aged SCs; thus, very few SCs break quiescence or engage in tissue repair [51]. In addition, aged SCs progressively express a high level of JAK/STAT signaling targets [53, 118], have elevated TGF-beta/pSmad2,3 [10], and perturbed p38 signaling [116, 117, 128–131], all of which promote myogenic differentiation at the expense of SC self-renewal and myoblast expansion. Similarly, the Wnt/beta-catenin pathway promotes the formation of fusion-competent myoblasts and myotubes, but also inhibiting the expansion of SCs when Wnt becomes excessive with age [8, 50].

### Tissue rejuvenation

**Fig. 3:**
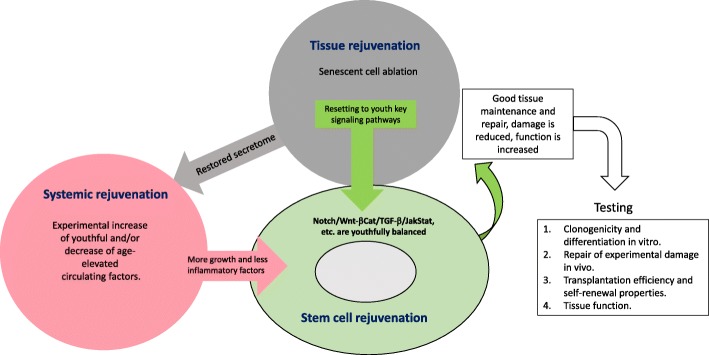
Summary of the approaches for tissue, systemic and stem cell rejuvenation. Multiple experimental approaches have been used (typically, in mice) for tissue rejuvenation and/or systemic rejuvenation; these include ablation of senescent cells and re-calibration of key signaling pathways that are needed for productive stem cell responses. To test the success in experimental rejuvenation, 1–4 approaches are typically applied, and skeletal muscle is well-suited for assaying each one, as described in the text

Muscle has served as an excellent model for assessing tissue rejuvenation because it undergoes clear-cut and well-described physiological, histological cellular and molecular changes with age. The summary of approaches for muscle rejuvenation is outlined in Fig. 3. In addition, adult myogenesis takes place throughout mammalian life and is well-characterized. At the beginning of muscle regeneration soon after the injury, small diameter myofibers with centrally located myonuclei are produced by the fusion of myoblasts. They can be distinguished histologically by morphology and expression of the embryonic/developmental isoforms of myosin heavy chain (eMyHC). With time (weeks), these myofibers increase in size and the myonuclei migrate to the periphery, so that regenerated muscles appear indistinguishable from undamaged muscles. A hallmark of the aging muscle is a decline in the formation of eMyHC+ myofibers after injury, persistence of inflammatory cells and cytokines, and expansion of fibrosis [132, 133].

An alternative method of assaying aging and rejuvenation consists of measuring the size of the new myofibers that repaired the injury, assuming that bigger myofibers are better. However, if the injury is successfully repaired by small muscle myofibers, there could have been prolonged myogenic proliferation at the expense of fusion or differentiation, and most myofibers eventually increase their size by fusing with each other and/or through protein synthesis and hypertrophy. The early time points after injury (5–7 days) serve best for assaying eMYHC+ centrally nucleated myofibers, for after 2 weeks, eMyHC expression is lost and regenerated myofibers begin to look similar to non-injured ones. However, for assaying the age-specific changes in muscle physiology and function, contractility, and strength, longer time points (2–4 weeks) are clearly preferable [52, 134].

The myogenic capacity of freshly isolated SCs can also be assayed in vitro by measuring the numbers of myoblast clusters that are produced in hours to days after derivation from the muscle and by the numbers and multi-nuclearity of myotubes that differentiate from those isolated myoblasts. In such assays, young SCs or myofibers with their associated SCs are typically more myogenic than their old counterparts. The age-specific changes in the clonogenic capacity have been studied in the muscle and are typical for other tissues such as hematopoietic, liver, bone, brain hippocampus, and skin, underscoring the significance of muscle as a superb experimental system in aging research. Linked to the clonogenic capacity and also generally shared by stem cells from different tissues is the age-specific transplantation efficiency of SCs [53, 62, 77, 95, 117, 135–140]. Interestingly, early muscle transplantation studies suggest that the age of the host rather than the age of the SCs seem to influence the success in regeneration [141].

Studying the above-described parameters in young, old, and experimentally rejuvenated muscle yielded a number of novel paradigms that broadly apply to tissue aging and rejuvenation [41, 42]. For example, experiments that allow sharing young donor constituents (blood, secreted molecules, and organs), with an old host, were shown to rejuvenate myogenicity and to restore the youthful Delta/Notch signaling after injury [8, 14, 142–144], but also rejuvenate the brain, cognition, liver, skin, bone, etc. Clinically relevant attempts to rejuvenate the circulatory niche of muscle stem cells include neutralization of Wnt and TGF-β in old mice by inhibiting the age-elevated ligand molecules and/or their signaling pathways [93, 145, 146]. Activation of FGF2-p38alpha/beta MAPK, ectopic oxytocin/MAPK, interleukin33 (IL33) supplementation, or IL6-JAK/STAT3 pathways, e.g., the determinants which decline with age, have also been shown to rejuvenate myogenic responses [147]. In a dual-prong approach, oxytocin (a signaling peptide that declines with age) was combined with a low dose of an inhibitor of TGF-beta/pSmad (signaling that increases with age). Emphasizing cross-tissue conservation of age-associated changes, this defined pharmacology not only enhanced muscle repair but also improved cognitive function through a probable reduction of neuroinflammation and reduced liver adiposity and fibrosis in old mice [148]. GDF11, once suggested as pro-regenerative youthful factor [142], was found to actually inhibit muscle regeneration [149] possibly through SCs inhibition [145]. The inhibitory role of GDF11 is consistent with the phenotypes of GDF11 gene knockout mice [146, 150] and the fact that this TGF-β family member activates pSmad 2, 3 signaling, which is already elevated in the old and well known to block cell proliferation in general and specifically of SCs [147, 149, 151]). A protein very similar to GDF11, myostatin (aka, GDF8) has a known inhibitory role for SCs proliferation and muscle growth; accordingly, its antagonist follistatin is pro-regenerative [152–154]. Like other TGF-β family proteins, GDF11 is pro-angiogenic and it might support muscle regeneration through increased blood vessel formation, albeit at risk of promoting oncogenesis, as GDF11 has a high association with human cancers [155–158].

### The age-associated biophysical and biochemical changes in the stem cell niche

The general directions of experimental rejuvenation are based on the fact that maintenance and repair of mammalian tissues is regulated by systemic and local cell signaling molecules [41, 42]. Skeletal muscle is a good example of the multi-level endocrine and local tissue control of homeostatic maintenance and regeneration. Muscle is highly vascularized, and the molecular composition of the systemic milieu has a profound influence on the maintenance and repair of this tissue. Heterochronic parabiosis and blood exchange (apheresis) studies uncovered the phenomenon of rapid restoration of regeneration in old muscle, through exposure to a young organism (in parabiosis) or just young blood (apheresis). These experiments pointed out the crucial age-specific roles for the SC niche, of interstitial cells, blood vessels, extracellular matrix proteins with their storage of secreted factors, as well as the systemic environment (circulation) for both the maintenance of SCs in the quiescent state and their activation for proliferation, differentiation, and tissue repair. In confirmation of the multi-tissue conservation of the paradigms uncovered in aged muscle, rejuvenation of the CNS, brain, bone, kidneys, liver, etc. have also been demonstrated through blood heterochronicity [41, 42]. Moreover, many key age-specific biophysical and biochemical changes that were established through studies of muscle apply more generally to these other tissues and clarify the overall age-imposed increases in fibrosis and inflammation.

Through its components (fibrillar proteins, growth factors, glycoproteins, chemokines, cytokines), the extracellular matrix (ECM) presents the biochemical and biophysical cues that home the SCs to specific locations of the myofiber and control the cell-intrinsic polarity and cell-fate decisions, which are essential for SC functionality [127, 159–161]. Laminin, the primary protein of the ECM, along with other glycoproteins such as type IV collagen, perlecan, entactin (nidogen), and fibronectin, support SCs proliferation [128–130, 162]. Proteoglycans act as receptors for precursor forms of growth factors (HGF, bFGF, EGF, IGF-I, IGF-II), which are required for activation of SCs in response to muscle damage [163, 164]. In return, SCs express the integrin receptors that interact with the basal lamina to regulate appropriate ECM deposition from fibroblasts and to prevent fibrosis [110, 165]. With age, muscle displays lower levels of elastin and fibronectin, which are cleaved and increasingly accumulate in the surrounding connective tissue, leading to compromised muscle maintenance and degradation of the ECM through tissue necrosis [166]. The age-imposed misprocessing of ECM proteins leads to the accumulation of toxic-by-products and altered properties of the basal lamina. Compromised interaction with the ECM also leads to weaker adhesion of SCs to their associated myofibers, and detachment or a perception of detachment leads to a programmed cell death called anoikis [130].

**Fig. 4:**
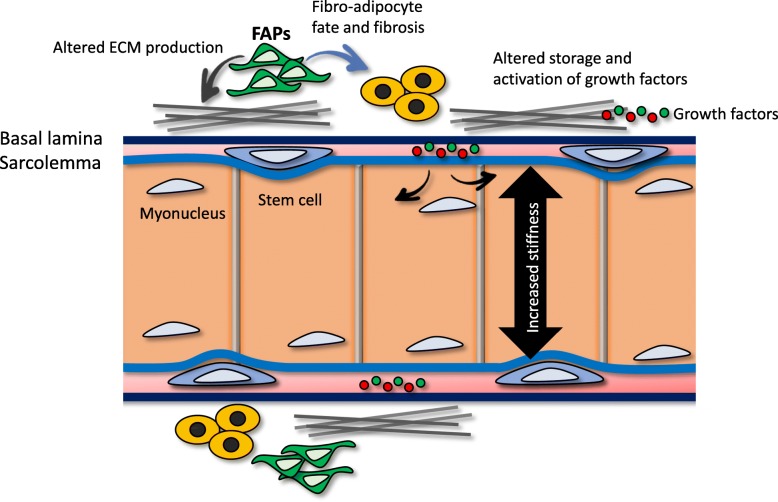
Connection between biochemical and biophysical age-associated tissue changes are exemplified in skeletal muscle. With age, composition of ECM becomes altered through changes in FAPs, persistent damage, fibrosis, and inflammation; these age-associated changes make myofibers stiffer and diminish the capacity of ECM for proper storage and activation of growth factors

ECM integrity and remodeling depends on the dynamic balance between remodeling enzymes (matrix metalloproteinases, MMPs) and their inhibitors (tissue inhibitors of metalloproteinases, TIMPs) [167, 168]. During muscle regeneration, MMP2 secreted by SCs and MMP9 produced by IL6 secreting leukocytes [169] degrade type IV collagen, among other constituents of the ECM, thereby allowing recruitment of activated SCs to the site of muscle injury [170]. In addition, MMP-9 converts the matrix-tethered latent TGF-β complex to an active form [171] and subsequently stimulates matrix deposition [172]. The persistent inflammation associated with aging leads to alterations in the composition of the ECM, where atypical types of collagen are seen along with a shift toward collagen IV and reduced collagen VI [173, 174]). The aged ECM retains fewer glycoproteins and is characterized by infiltration of adipose and fibrotic tissues [8, 87]. Together, these age-imposed processes ultimately drive an increase in fibrosis and matrix rigidity, increasing the elastic modulus to ∼ 418 kPa instead of the productive ∼ 12 kPa of the young muscle [72]. Aged single myofibers also have an increased physical stiffness that correlates with the increased crosslinking of their collagens [175, 176], and when cultured on hydrogels that mimic this stiffness, adult primary myoblasts show increased differentiation at the expense of proliferation [175]. The deposition of extra basal lamina into the SC-myofiber interspace interferes with the intimate association between SC and their myofibers [103]. This expulsion from the niche changes multiple molecular cues that regulate the asymmetry of SC divisions and their cell-fate, and it might cause the disparity in young versus old SC cell counts between bulk fiber preparations as opposed to single fiber studies [8]. In addition, with age, the ability of the ECM to function as a reservoir for growth factors and their conversion to active forms become altered [174]. Age-imposed changes in the ECM composition perturb regeneration through inadequate support of muscle fibers and disorganized scaffold orientation [177–179]. The p38α/β MAPK axis was shown to play an essential role in muscle mechanobiology [117, 130], and age-imposed changes in muscle tensegrity contribute to the impaired function of SCs [149, 175, 176, 180]. The main age-specific changes in muscle ECM are depicted in Fig. 4.

In concert with the studies in muscle, work with other cell types (including mammary epithelial, fibroblasts and mesenchymal stem cells) indicates significance of age-specific changes in ECM for loss of stem cell properties and accumulation of senescent cells and suggests that interactions between integrin-focal adhesion complexes and the actin-myosin network broadly help cells to sense matrix elasticity, which in turn influences cell behavior and fate [181–186].

### Age-specific changes in the epigenome

The environment largely influences the epigenomic program (i.e., post-translational modifications), which determines the fate of activated adult stem cells through the expression or repression of specific genes. Studies of muscle have greatly contributed to the broad understanding of age-associated epigenetic changes in stem cells. Namely, the changes that were observed between young versus old SCs and were correlated with the global transcriptome of these stem cells [53, 116, 187] have been extrapolated to other tissues and their stem cells, such as hematopoietic [188], heart [188], and brain [188, 189].

Notch signaling might contribute to the age-imposed changes in the SC epigenome through positive regulation of Bmi1 [96, 190, 191], a component of the polycomb repressive complex 1 (PRC1), in coordination with enhancer of zeste homolog 2 (Ezh2), a component of PRC2. Together, they repress the expression of several genes such as p16^INK4a^ through maintenance of H3K27me3 marks [192, 193]. With age, the redistribution of PRC1 and PRC2 may activate SCs and inhibit their self-renewal, driving a cellular senescence phenotype associated with aged SCs [194–197]. Evidence of this pathway comes from the observation that deletion of Bmi1 in young SCs prevents their active participation in muscle regeneration [197]. Similarly, elevated with age TGF-beta and diminished MAPK signaling activate the expression of CDK inhibitors and promote cell cycle arrest in muscle SCs and in neural precursor cells [10, 84, 198].

Some studies on epigenetic and transcriptional profiling in SCs suggest that the overall permissive state (e.g., H3K4me3) is age unrelated. However, the dominant and repressive marks (e.g., H3K27me3) accumulate and spread with age [187], probably reflecting the decrease in proliferative capacity and the inability of SC to self-renew as these repressive epigenetic marks are transmitted to daughter cells [194–197]. An age-imposed loss of epigenetic inactivation of CDK inhibitors loci takes place in SCs, resulting in permissiveness of CDK expression and a lack of myogenic proliferation [84, 96]. Aged activated SCs also display an altered epigenetic stress response [199]. Interestingly, the experimental activation of FGF2/MAPK reverses the age-imposed epigenetic anti-proliferative signature to a younger, closed chromatin state [84, 200].

In this regard, there is an observation of a very slow and gradual SC exhaustion though proliferation that might be relevant for old people, particularly those who constantly damage muscle by extremely rigorous exercise [93, 201]. However, in mice, virtually, no SCs incorporate BrdU in uninjured muscle and are mitotically quiescent in the young (2 months or older) or the old animals [62, 202, 203]. Nevertheless, even in the absence of SC exhaustion, mouse muscle ages (much faster than that of humans) with pronounced lack of SC responses and sarcopenia. Moreover, all examined CDK inhibitors (p15, p16, p21, p27) become elevated in aged SCs, as compared to young, and there is an age-imposed epigenetic permissiveness of the p16^INK4a^ and p21^CIP1^ loci in old SCs after injury [84]. With age, there is a loss of the PRC1-mediated repressive H2A-lysine 119 ubiquitination mark, which leads to de-repression of the p16^INK4a^ locus and SC inactivation, a loss of myogenic fate (absence of MyoD), and the acquisition of a senescent cell fate that is marked by elevated gamma-H2A histone family member X (γH2AX) foci and secretion of the “senescence-associated secretory phenotype” [96].

Generally speaking, the lack of activation versus too much activation (e.g., proliferative exhaustion are the general paradigms under investigation in the broad area of tissue stem cell aging) and the understanding of these phenomena in muscle resonates well with the work in the gut, skin, blood, and brain [119, 204–208].

### Inflammation

As true in other tissues that undergo life-long remodeling (gut, bone, blood, liver, skin, etc. [209–211]), muscle regeneration and inflammation coincide in space and time [212]. The study of muscle provided insights into the process of the age-specific decline in tissue maintenance and the dominance of inflammation. To some degree, inflammation is useful for tissue repair: the inflammatory response, mostly by myeloid cells, removes the degenerating debris, and the temporary scar allows the correct orientation and deposition of new ECM by muscle-resident fibroblasts, which also provide pro-differentiation signals to myoblasts. Some inflammatory cytokines and myokines are produced and promote myogenesis, activate endothelial cells for angiogenesis, and attract new nerve projections [88, 213–217].

Numerous immune cells infiltrate damaged muscle, with neutrophils being the first responders to the injured site at 1–6 h. These secrete pro-inflammatory molecules such as cytokines (TNF-alpha, IL6), chemokines (CCL2 and 17), and growth factors (FGF, HGF, IGF1; VEGF, TNF-beta) that create a chemo-attractive environment to monocytes and macrophages. M1 phagocytic CD68+/CD163− macrophages arrive at 2 days post-injury and are replaced by M2 non-phagocytic CD68−/CD163+ macrophages at 4 days post-injury [218, 219]. This switch in the macrophage populations has been described as critical for stopping inflammation and enabling both the differentiation and fusion of myoblasts [220, 221]. With aging, the M1 profile dominates over M2 during muscle repair [222, 223], which is in part due to the elevation of macrophage-produced osteopontin, which in turn induces a battery of inflammatory cytokines that inhibit myogenesis [87] and phagocytic activity. The M1 to M2 switch that was found in studies of skeletal muscle is a general trend with aging and is responsible to diminished repair and increased chronic inflammation in the joints, lung, liver, the gastrointestinal track, and other tissues. Recently, another class of immune cells, T regulatory cells (Tregs), has gained interest due to their ability to dampen the inflammatory response and promote tissue repair [224] in the muscle, heart, skin, kidney, and brain [225–229]. In aged muscle, the lack of local secretion of IL33, probably by the fibro-adipogenic progenitor (FAP)-like cells (the major source of this inflammatory cytokine), impairs the attraction of Tregs to the injury site, and results in a decline of regenerative capacity [230].

Age-elevated inflammation negatively impacts not only SCs [112–114], but also other cell types, mostly stromal cells, such as blood vessel associated mesoangioblasts, mesenchymal stem cells, FAPs, ALDH+/CD34- cells, CD133+ cells, and pericytes [231–238]. Most of these have been studied in age-comparative ways in muscle [239–241] and are clearly important for most mammalian tissues. Of particular interest, FAPs constitute a non-myogenic population essential for muscle regeneration. Undifferentiated quiescent FAPs in the interstitium of healthy young muscle have positive effects on SCs activation and the proliferation of myoblasts, potentially via secretion of IL6, IGF1, Wnt1, Wnt3a, and Wnt5a [238, 242]. However, excessive activation of FAPs following injury in aged muscle induces their differentiation into adipocytes and into the myofibroblasts that are the main secretors of type I collagen and contribute to progressive fibrosis. Fibrosis is further promoted in old muscle through activation of adipocytes when eosinophil production of IL4 declines [243], and the cytokine profile of macrophages becomes pro-inflammatory [244].

### Selecting a specific sample size in studies of aging

Considering recent focus on scientific rigor and the large variety of approaches in muscle aging research, this review will end with a section on one key scientific parameter—sample size—providing out perspective on choosing the optimal numbers of experimental animals. Researchers investigating aging and rejuvenation of muscle and other tissues typically experiment on 5–6 male mice per cohort, and historically, these numbers yielded statistically relevant, robust data [7, 8, 51, 115]. However, some report as many as 10–15 animals per cohort [109, 142]. So how many animals are really needed?

The size and the composition of the cohorts are crucial as they determine the relevance of the observed effects, while attempting to comply with ethical considerations and limitation in the use of resources. The National Research Council’s Guide for the Care and Use of Laboratory Animals guidelines state that the number of live animals used for research should be minimized. The tenets of ethical animal use are described as “the three R’s”: replacement, refinement, and reduction [245]. The reduction principle aims to maximize the amount of data collected from the fewest number of animals practical.

Due to the law of diminishing returns [246], having an unnecessarily large sample size results in negligible gains in statistical significance that do not justify extra costs, animals, or time. Inversely, selecting too small a sample size runs the risk of the experiment having inadequate power for detecting significant effects, which also renders the financial, animal, and time resources wasted [246–250]. Ideally, the sample size should be sufficiently large to provide the experiment with adequate statistical power, while at the same time minimizing the number of animals needed to achieve statistically significant results. The method used for accurate determination of the sample size primarily depends on whether there are existing data to inform a prediction of the treatment effect size, ES, and the population standard deviation, σ. Statistical power analysis is the most robust method for determining sample size, and it is used whenever at least some population statistics are available. When no prior statistics are available to do a power analysis, a pilot study is done using a resource equation to determine the number of animals needed to detect any effect of an exploratory condition. This scenario could be minimized by searching the literature for population data that could be used for a power analysis. The key aspects of the power analysis and resource equation are briefly outlined below.

Generally speaking, when a normally distributed population mean and standard deviation can be reasonably estimated, and it can be assumed that the experimental data will be normally distributed, then statistical power analysis is used to determine the minimum number of animals *n* per cohort. In such analysis, the null hypothesis *H*_0_ and the alternative hypothesis *H*_*A*_ are defined as follows:

$$ {H}_0:\overline{X}=\mu $$

$$ {H}_A:\overline{X}\ne \mu $$

where *μ* is the presumed population mean, and $\overline{X}$ is the sample mean. Rejecting the null hypothesis when the sample mean is not different from the population mean results in a type I error and occurs with probability *α*. Failing to reject the null hypothesis when the sample mean truly differs from the population mean results in a type II error and happens with probability *β*. This is summarized [247] and depicted in Table 1.

**Table 1:** Outcome space of a hypothesis test

	$\overline{X}=\mu$	$\overline{X}\ne \mu$
Reject *H*_*0*_	Type I error	Correct conclusion
Accept *H*_*0*_	Correct conclusion	Type II error

The power of a hypothesis test is the probability of rejecting *H*_0_ when it is indeed false. This is simply the complementary probability to *β* or making a type II error:

$$ \mathrm{Power}=1-\beta $$

**Fig. 5:**
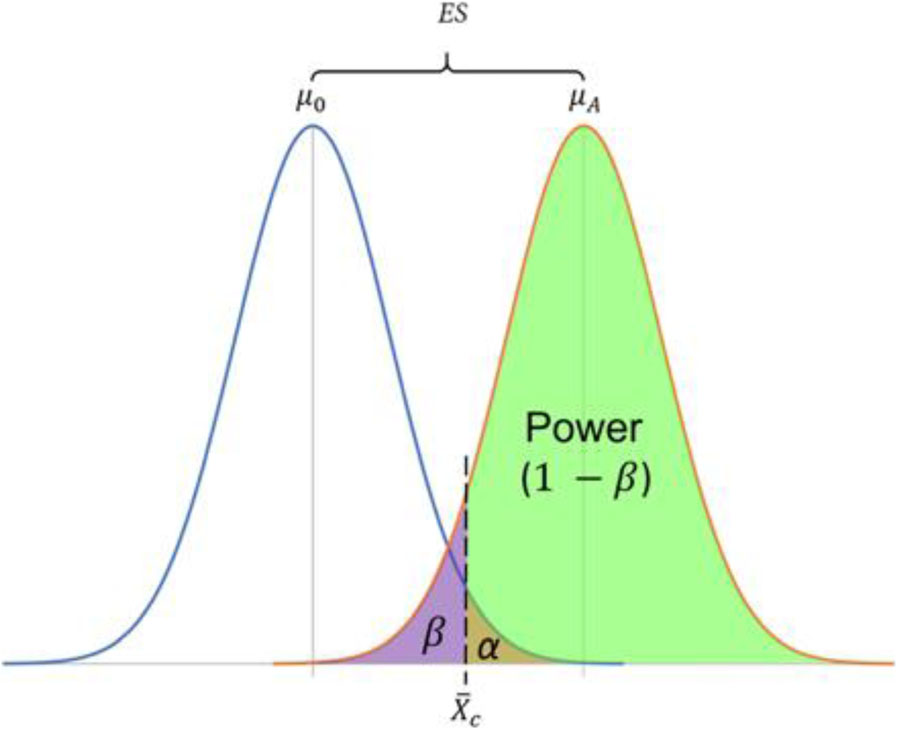
The relationship between ES, *α*, *β*, and power for a one-tailed test where it is expected that *μ*_*A*_ > *μ*_0_. The critical value ${\overline{X}}_c$ is the minimum sample mean to needed to reject *H*_0_ at the desired confidence level (1 − *α*). Note that for a given *α* and ES, the area of *β* increases and the power decreases with increasing variability in the distributions. Conversely, if variability decreases, the power increases and *β* decreases

The probability *β*, and therefore the power, depends on *α*, the sidedness of the test (one-tailed or two-tailed), the effect size ES of the treatment, *σ*, and the sample size *n*. From this relationship, one solves for the minimum *n* needed to detect a desired ES with a test having a desired confidence level and statistical power. The interplay between ES*, α, β* and other parameters is visualized in Fig. 5 [247–251].

In general, as the desired confidence level for the test increases, the probability of a type I error decreases, but at the expense of power. Decreases in power and/or confidence can be mitigated by a tight distribution of the data (low *σ*), a large ES, or by increasing *n* (which has the effect of lowering *σ*). However, in adhering to the reduction principle, *n* should be minimized by some combination of decreasing our confidence, decreasing the power, or increasing the minimum ES detectable by the test. Typical acceptable values for *α* are 0.05 or lower, and typical values for power are 0.8 or 0.9.

There are numerous online calculators to determine sample size such as:

https://www.stat.ubc.ca/~rollin/stats/ssize/n2.html

https://www2.ccrb.cuhk.edu.hk/stat/mean/osm_equivalence.htm

Finally, to ensure the success of the experiment, the researcher must account for the expected attrition rate *A* (in particular working with old mice, some may die from “old age” during the experiment) and calculate the corrected sample size *n*^′^ [11]:

$$ {n}^{\prime }=\frac{n}{1-A} $$

For exploratory treatments where there is no reliable a priori knowledge to inform about the effect size or standard deviation, a power analysis to determine sample size is not feasible. A pilot study can be done, not to measure actual effect size, but rather to determine if there is any detectable difference between control and experimental groups. To adhere to the reduction principle, the number of animals should still be minimized in pilot studies, but a sufficiently large sample size is also needed for adequate detection power. A resource equation can be used to infer the smallest sample size that is nevertheless adequate to detect variability between groups [249–253].

An experiment with sample size *N* testing for the effects of a treatment can have at most *N* − 1 degrees of freedom (df) or points allowing for variability [252]. The resource equation breaks this variability into three components: blocking *B = b* − 1, treatment *T* = *t* − 1, and error *E* dfs. Blocking refers to the separation of cohorts into *b* groups based on environmental factors (or, sex, age, etc.). *T* refers to the number of questions *t* being asked. *E* is used as an estimation of the variance within treatment groups. The total (*N* − 1) df is equal to the sum of the dfs of the three variability components:

$$ B+T+E=N-1 $$

For a good estimate of the variance, *E* must be greater than 10, but for values greater than 20, there is a negligible gain in statistical significance which would not justify the increased number animals. With that in mind, it is up to the researcher to decide on the value of *E* when solving for *N*.

Using higher numbers of animals than those suggested by the above resource equation or power analysis have been concluded not to yield better or more reliable data, and indeed, high sample numbers did not overcome conflicting results in comparative body of published work on GDF11 and pSMAD signaling and aging. In our experience, if a small number of animals per cohort do not show a robust difference between experimental and control groups, then perhaps the researcher should consider a more robust experimental assay or a different experimental approach to answer the question. We also find multiple experimental approaches, each with smaller cohorts, to answer the same general question to be a more rewarding use of time and resources. For example, two experiments, one examining the effects of modulating a ligand and another modulating the receptor or downstream signaling, will give either corroborating or conflicting results, and that depends more on whether the phenomenon is robust or not and less on how many animals were used in the assays. Finally, the bulk of studies on muscle aging and rejuvenation are mostly if not only from male mice that, moreover, are genetically identical and environmentally similar. Therefore, the magnitude of effects and robustness should be interpreted with caution as they may not translate exactly to clinical studies [254].

## Conclusion

In recent decades, the health and regeneration of skeletal muscle have been frequently used as key experimental systems in studies that focused on understanding and reversing mammalian tissue aging. This body of work enriched the field of adult myogenesis, the broader arena of aging research, and yielded advances in stem cell isolation and characterization, pathway reconstruction, omics, etc. biomedical approaches. The field of muscle research in general and in application to aging is still burgeoning as revealed by innovative technologies and exemplified by in situ single-cell cartography, the high definition comprehensive mapping of muscle resident types [255]. Aging research in muscle is multi-disciplinary, and it cross-pollinates different fields of science, including stem cell biology and regenerative medicine, bioengineering and mechanobiology, Big Data, omics, and imaging. Such diversity of technologies and approaches enables robust and rigorous checks and validations of the findings by the body of published work in this clinically relevant field of science, ultimately yielding feasible therapies for extending productive health span.

## Data Availability

Not applicable

## Abbreviations

ALDH: Aldehyde dehydrogenases
bFGF: Fibroblast growth factor-basic
BrdU: Bromodeoxyuridine
CCL2/17: Chemokine ligand 2/17
CD 33/45/68/163: Cluster of differentiation 33/45/68/163
CDKIs: Cyclin-dependent kinase inhibitor protein
c-Met: Tyrosine-protein kinase Met
CNS: Central nervous system
Coll: Collagenase
CXCR4: C-X-C chemokine receptor type 4
Df: Degree of freedom
DMEM: Dulbecco’s modified Eagle medium
DNA: Deoxyribonucleic acid
ECM: Extracellular matrix
EDL: Extensor digitorum longus
EGF: Epidermal growth factor
eMYHC: Embryonic myosin heavy chain
Ezh2: Enhancer of zeste homolog 2
F-10: Ham’s F-10 Nutrient Mixture
FACS: Fluorescence-activated cell sorting
FAPs: Fibro-adipogenic progenitors
FBS: Fetal bovine serum
FGF: Fibroblast growth factors
Gamma-H2AX or γH2AX: Gamma-H2A histone family member X
GDF8/11: Growth differentiation factor 8/11
H3K27me3: Tri-methylation at the 27th lysine residue of the histone H3 protein
H3K4me3: Tri-methylation at the 4th lysine residue of the histone H3 protein
HGF: Hepatocyte growth factor
IGF1: Insulin-like growth factor 1
IL4/6/33: Interleukin 4/6/33
ITGB1: Integrin beta 1
JAK: Janus kinase
kPA: Kilo pascal
M1/2: Macrophage type M1/M2
MAPK: Mitogen-activated protein kinase
microRNA: Microribonucleic acid
MMP: Matrix metalloproteinases
Myf5: Myogenic factor 5
MyoD: Myoblast determination protein 1
NCAM: Neural cell adhesion molecule
p15: Cyclin-dependent kinase 4 inhibitor B (CDKN2B)
p16^INK4a^: Cyclin-dependent kinase inhibitor 2A (CDKN2A)
p21^Cip1^: Cyclin-dependent kinase inhibitor 1 (CDKN1A)
p27: Cyclin-dependent kinase inhibitor 1B (CDKN1B)
Pax3/7: Paired box gene 3/7
PRC1/2: Polycomb repressive complex 1/2
ROS: Reactive oxygen species
SC: Satellite cells
Sca1: Stem cells antigen 1
STAT3: Signal transducer and activator of transcription 3
TGF-beta: Transforming growth factor beta;
TIMPs: Tissue inhibitors of metalloproteinases
TNF-beta: Tumor necrosis factor-beta
Tregs: Regulatory T cells
VCAM: Vascular cell adhesion protein 1
VEGF: Vascular endothelial growth factor
WNT1/3a/5a: Wingless-related integration site1/3a/5a
